# Aurora-A promotes chemoresistance in hepatocelluar carcinoma by targeting NF-kappaB/microRNA-21/PTEN signaling pathway

**DOI:** 10.18632/oncotarget.2682

**Published:** 2014-11-04

**Authors:** Kai Zhang, Jing Chen, Dongqin Chen, Jiayuan Huang, Bing Feng, Siqi Han, Yitian Chen, Haizhu Song, Wei De, Ziman Zhu, Rui Wang, Longbang Chen

**Affiliations:** ^1^ Department of Medical Oncology, Jinling Hospital, School of Medicine, Nanjing University, Nanjing, Jiangsu, China; ^2^ Department of Biochemistry and Molecular Biology, Nanjing Medical University, Nanjing, Jiangsu, China; ^3^ Department of Hepatobiliary Surgery, First Hospital Affiliated to the Chinese PLA General Hospital, Fucheng, Haidian District, Beijing, China

**Keywords:** Hepatocellular carcinoma, Aurora-A, NF-kappaB, MicroRNA-21, PTEN, Chemoresistance, Apoptosis

## Abstract

Hepatocellular carcinoma (HCC) is highly resistant to chemotherapy. Previously, we have shown that Aurora-A mRNA is upregulated in HCC cells or tissues and silencing of Aurora-A using small interfering RNA (siRNA) decreases growth and enhances apoptosis in HCC cells. However, the clinical significance of Aurora-A protein expression in HCC and association between Aurora-A expression and HCC chemoresistance is unclear. Here, we showed that Aurora-A protein is upregulated in HCC tissues and significantly correlated with recurrence-free and overall survival of patients and multivariate analysis indicated that immunostaining of Aurora-A will be an independent prognostic factor for patients. Silencing of Aurora-A significantly increased the chemosensitivity of HCC cells both *in vitro* and *in vivo*, while overexpression of Aurora-A induced the opposite effects. Furthermore, overexpression of Aurora-A reduces chemotherapy-induced apoptosis by promoting microRNA-21 expression, which negatively regulates PTEN and then inhibits caspase-3-mediated apoptosis induction. Mechanically, we demonstrated that Aurora-A promotes expression of nuclear Ikappaβ-alpha (Iκβα) protein and enhances NF-kappa B (NF-κB) activity, thus promotes the transcription of miR-21. This study first reported the involvement of Aurora-A/NF-κB/miR-21/PTEN/Akt signaling axis in chemoresistance of HCC cells, suggesting that targeting this signaling pathway would be helpful as a therapeutic strategy for the reversal of chemoresistance in HCC.

## INTRODUCTION

HCC is the fifth most common cancer around the world with approximately 564,000 new cases diagnosed every year, and over 40 percent of all cases of HCC occur in China, which has an annual incidence of 137,000 cases [1]. Although surveillance can lead to early diagnosis when the tumor might be resectable, most patients present at an advanced stage when operation is no longer feasible and can only receive palliative treatments. Systemic or selective intra-arterial administration of any chemotherapy agent has been a common treatment modality for inoperable HCC, but it is far from satisfaction due to the intrinsic and acquired resistance of HHC cells to chemotherapeutic agents [2]. Therefore, it is needed to explore the molecular mechanisms involved in HCC chemoresistance in order to exploit suitable chemosensitizers for HCC patients.

Aurora kinases are a novel family of serine / threonine kinases which promote mitotic spindle assembly by regulating centrosome duplication and separation [3]. Three Aurora kinases (Aurora-A, Aurora-B and Aurora-C) have been identified, and the three molecules share a high affinity of amino acid sequence, but their subcelluar localization and functions are quite distinct. Aurora A (also named Aurora 2, STK15, BTAK, mouse Stk6, or Iak1) was first identified as a human homologue of the Aurora/Ipl1p kinase family, which is located at chromosomal region 20q13.2 and contains a 1209-bp open reading frame that encodes 403 amino acids with a molecular weight of 46 kDa [4]. Aurora-A is essential for mitosis and plays an important role in tumorigenesis and tumor development. It has been reported that ectopic expression of Aurora-A in NIH3T3 and Rat1 fibroblasts results in abnormal centrosome amplification and cellular transformation [5]. Meanwhile, overexpression of Aurora-A has been detected in a variety of human malignancies, such as breast cancer, esophageal squamous cell carcinoma (ESCC) and bladder cancer, etc [6-8]. In our previous study, we have shown that status of Aurora-A mRNA expression might be a good marker for predicting the prognosis of HCC patients and siRNA-mediated Aurora-A downregulation could lead to growth inhibition and apoptosis enhancement in HCC cells both *in vitro* and *in vivo* [9,10]. However, the roles of Aurora-A in chemoresistance of HCC cells and the possible molecular mechanisms are unclear and remain to be further elucidated.

In this study, we first performed Western blotting and immunohistochemistry assays to detect the expression of Aurora-A protein and analyze its clinicopathological or prognostic significance in human HCC. Also, we investigated how Aurora-A regulates chemoresistance in human HCC cells. Specifically, we determined the role of the NF-κB/miR-21/PTEN signaling in affecting the chemosensitivity of HCC cells by regulating the ratio of Bcl-2/Bax and the activation of the mitochondrial apoptotic pathway. Our results indicated that positive Aurora-A protein expression in HCC tissues was significantly correlated with poorer RFS and OS of patients, and Aurora-A promotes *in vitro* and *in vivo* chemoresistance of HCC cells by reducing chemotherapy-induced apoptosis via activation of NF-κB/miR-21/PTEN signaling pathway. Therefore, overexpression of Aurora-A plays critical roles in HCC progression and chemoresistance, and targeting Aurora-A/NF-κB/miR-21/PTEN signaling will be a promising strategy for chemosensitization of human HCCs.

## RESULTS

### The expression of Aurora-A protein is upregulated in HCC tissues and correlated with HCC progression

**Figure 1:**
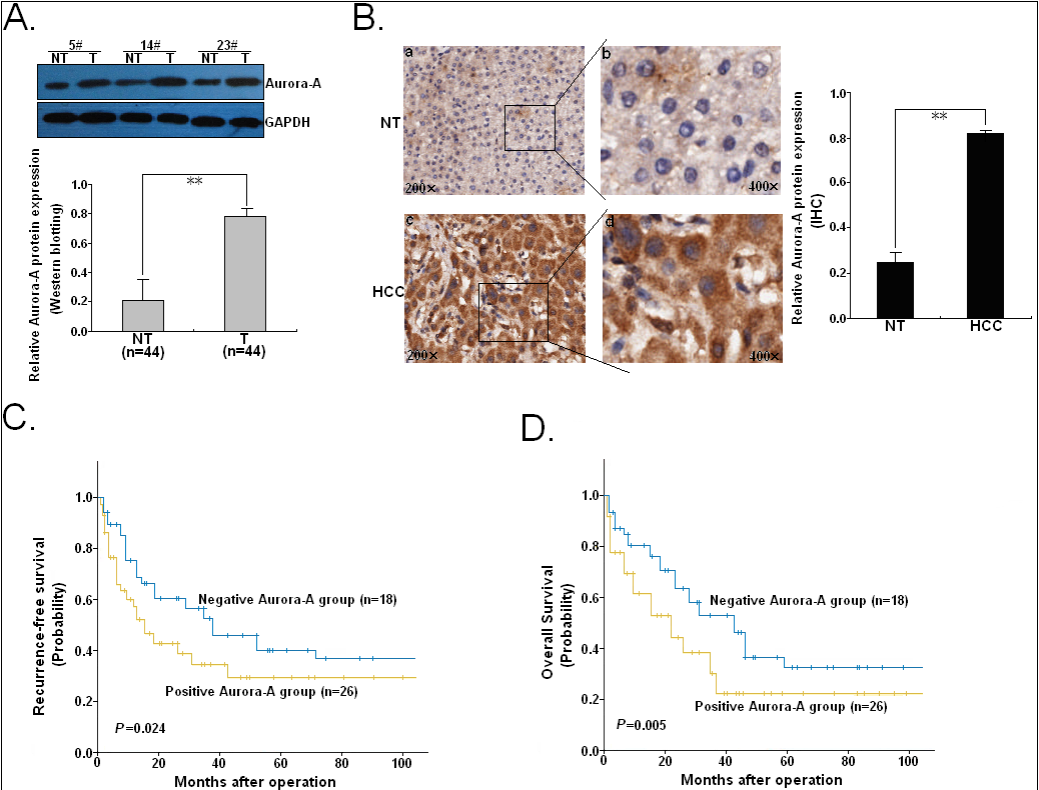
Expression of Aurora-A protein in HCC tissues and its correlation with prognosis of patients (A) Western blotting was performed to detect Aurora-A protein level in 44 paired of HCC and NTs. Three pairs of human HCC samples were exhibited. GAPDH was used as an internal control. (B) Immunohistochemical analysis. Intensive staining was observed in the cytoplasma of HCC cells, whereas less staining was showed in NTs (original magnification a, c: ×200; b, d: ×400). (C) The Kaplan-Meier survival curve of recurrence-free survival (RFS) according to Aurora-A immunostaining in 44 cases of HCC patients. (D) The Kaplan-Meier survival curve of overall survival (OS) according to Aurora-A immunostaining in 44 cases of HCC patients. Data were presented as mean ± SD of at least three independent experiments. *N.S, P*>0.05; *P<0.05; **P<0.01.

Previously, we have shown that the expression of Aurora-A mRNA is significantly upregulated in HCC tissues and correlated with poor patients' prognosis, but status of Aurora-A protein expression and its roles in HCC development are unclear. Thus, Western blotting and immunohistochemistry assays were performed to detect protein level and significance of Aurora-A in 44 pairs of primary HCC and corresponding nontumor liver tissues (NTs). Western blotting analysis revealed that Aurora-A protein was upregulated in HCC tissues compared with paired NTs (Figure 1A). Also, the increased expression of Aurora-A protein was observed in 32 (72.7%) HCC tissues compared with only 8 (18.2%) NTs (Supporting Table 1; *P*<0.001). Meanwhile, the expression of Aurora-A protein was determined by immunostaining assay, and the results indicated that 26 (59.1%) HCC tissues were positively stained, whereas only 12 (27.3%) NTs were positive for Aurora-A (Supporting Table 2, Figure 1B; *P*<0.001). Correlative analysis of positive Aurora-A protein expression with clinicopathological characteristics suggested significant correlations between increased Aurora-A expression and the increased number of tumor nodule and advanced tumor stages (Supporting Table 3). Furthermore, overexpression of Aurora-A in HCC was also found to predict the poorer RFS (*P*=0.024) and OS (*P*=0.005) of patients (Figure 1C and D). In a multivariate Cox model that included gender, age, Alchohol intake, liver function, AFP, TNM stage, lymph node metastasis, edmondson grade and Aurora-A protein immunostaining, we found that positive Aurora-A protein expression independently indicated poor prognosis for both 5-year RFS (HR: 2.055; 95% CI: 1.735-3.455; *P*=0.012) and 5-year OS (HR: 1.554; 95% CI: 1.232-2.598; *P*=0.005) in HCC patients (Supporting Table 4). Taken together, the protein expression levels of Aurora-A, which was significantly higher in HCC tissues than in NTs, plays an important role in HCC development.

### Cytotoxicity of ADR or CDDP in HCC cell lines and effects of ADR or CDDP on the expression of Aurora-A and apoptosis-related proteins

**Figure 2:**
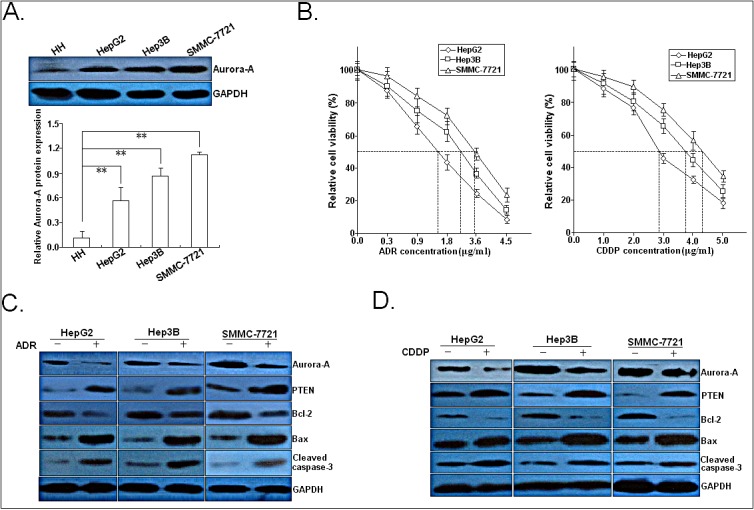
Effects of chemotherapy on the expression of Aurora-A and apoptosis-related proteins (A) Western blotting detection of Aurora-A protein expression in three HCC cell lines (HepG2, Hep3B and SMMC-7721) and a normal human hepatocyte cell line (HH). (B) MTT analysis of IC_50_ values of ADR and CDDP in three HCC cell lines (HepG2, Hep3B and SMMC-7721). (C) 24h after HCC cells (HepG2, Hep3B and SMMC-7721) were treated with ADR (0.5 μg/ml), Western blotting detection of Aurora-A, p-Aurora-A, PTEN, Bcl-2, Bax and cleaved caspase-3 proteins. (C) 24h after HCC cells (HepG2, Hep3B and SMMC-7721) were treated with CDDP (1.0 μg/ml), Western blotting detection of Aurora-A, p-Aurora-A, PTEN, Bcl-2, Bax and cleaved caspase-3 proteins. GAPDH was used as an internal control. Data were presented as mean ± SD of at least three independent experiments. *N.S, P*>0.05; *P<0.05; **P<0.01.

To determine the expression of Aurora-A in HCC cell lines, Western blotting was performed to detect the expression of Aurora-A protein in a panel of HCC cell lines (HepG2, SMMC-7721, Hep-3B) and a normal human hepatocyte cell line (HH). As shown in Figure 2A, the expression levels of endogenous Aurora-A protein were higher expressed in all of the three HCC cell lines than in HH cell line. The cytotoxic effect of ADR and CDDP was examined in three HCC cell line (HepG2, SMMC-7721 and Hep-3B), and we observed that SMMC-7721 and Hep3B cells which both showed high level of Aurora-A expression seemed to be more resistant to higher concentrations of ADR or CDDP than HepG2 cells which showed low level of Aurora-A (Figure 2B). Then, we further determined expressions of phospho-Aurora-A (p-Aurora-A), total Aurora-A and other apoptosis-related proteins (PTEN, Bcl-2, Bax and cleaved caspase-3) after ADR or CDDP treatment of HCC cell lines, and results indicated that pretreatment with ADR (0.5 μg/ml) or CDDP (1.0 μg/ml) for 24h led to decrease in expression of Aurora-A, p-Aurora-A and Bcl-2 proteins, and increase in expression of Bax, PTEN and cleaved caspase-3 proteins (Figure 2C and D). These data suggest that there existed a functional linkage among Aurora-A, PTEN and other apoptosis-related proteins in the formation of chemoresistance in HCC cells.

### Silencing of Aurora-A increases *in vitro* and *in vivo* chemosensitivity of HCC cells by enhancing chemotherapy-induced apoptosis

**Figure 3:**
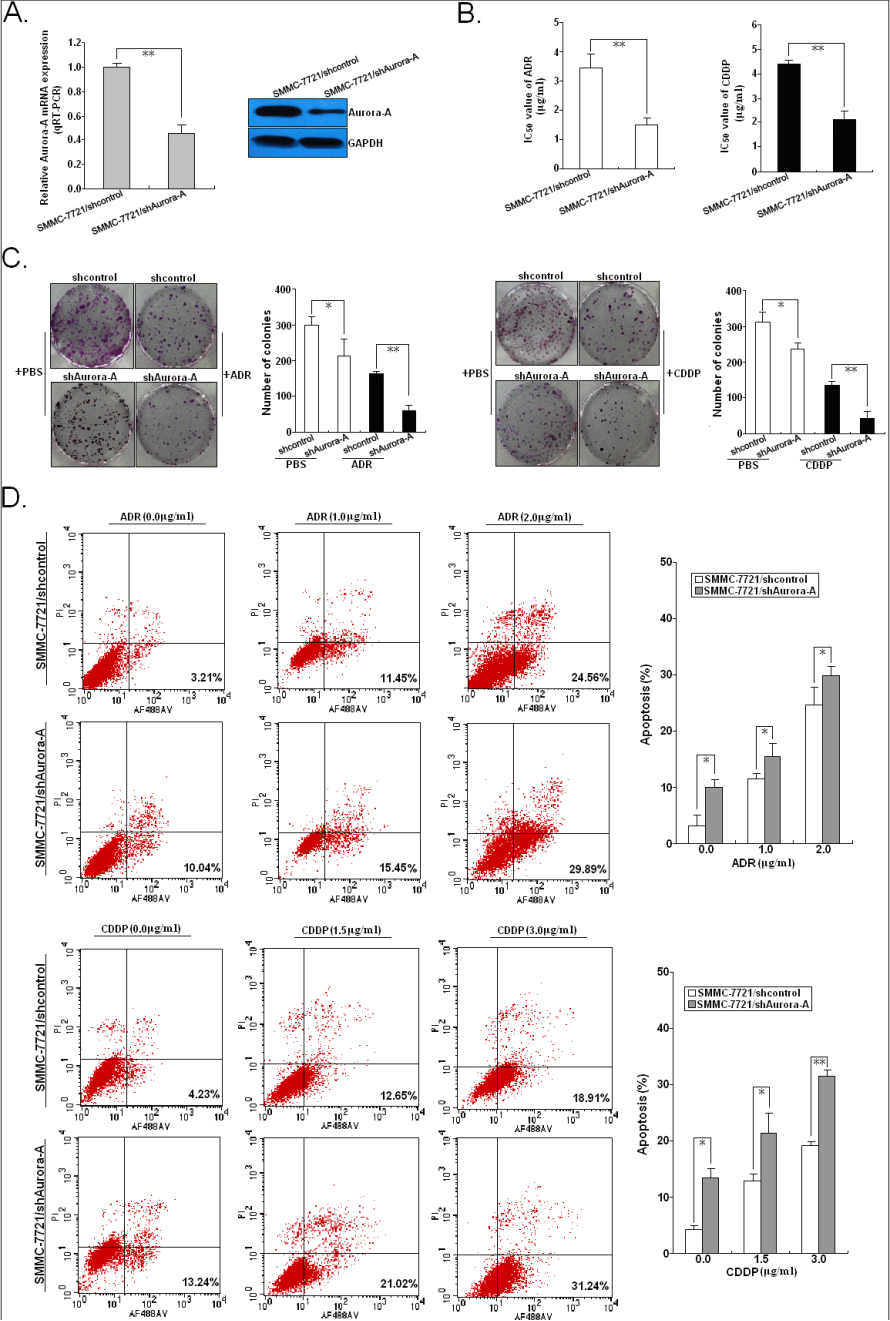
Effects of Aurora-A downregulation on *in vitro* chemosensitivity of HCC cells (A) qRT-PCR and Western blotting detection of Aurora-A mRNA and protein expression in stably transfected SMMC-7721/shAurora-A or SMMC-7721/shcontrol cells, respectively. GAPDH was used as an internal control. (B) MTT analysis of IC_50_ values of ADR and CDDP in SMMC-7721/shAurora-A or SMMC-7721/shcontrol cells, respectively. (C) The colony formation of SPC-A1 and SPC-A1/DTX cells treated with various concentrations of ADR (0.0 or 0.5 μg/ml) or CDDP (0.0 or 1.0 μg/ml). (D) Flow cytometric analysis of apoptosis in SMMC-7721/control or SMMC-7721/Aurora-A cells combined with various concentrations of ADR (0.0, 1.0 and 2.0 μg/ml) or CDDP (0.0, 1.5 and 3.0 μg/ml). Data were presented as mean ± SD of at least three independent experiments. *N.S, P*>0.05; *P<0.05; **P<0.01.

To determine whether downregulation of Aurora-A affected the sensitivity of HCC cells to chemotherapeutic agents (ADR and CDDP), SMMC-7721 cells was stably transfected with pSil/shAurora-A or pSil/shcontrol, respectively. qRT-PCR and Western blotting assays confirmed the depletion of endogenous Aurora-A in SMMC-7721 cells (Figure 3A). The results indicated that the IC_50_ values of both ADR and CDDP were significantly reduced by Aurora-A downregulation in SMMC-7721 cell line (Figure 3B). The IC_50_ value of ADR or CDDP in SMMC-7721/shAurora-A cells was 1.48±0.32 or 2.15±0.56 μg/ml (*P*<0.01), respectively, while the IC_50_ value of ADR or CDDP in SMMC-7721/shcontrol cells was 3.45±0.67 or 4.42±0.26 μg/ml, respectively. Then, we determined the effect of Aurora-A downregulation on colony formation ability of HCC cells when exposed to ADR (0.0 or 0.5 μg/ml) or CDDP (0.0 or 1.0 μg/ml) treatment. As shown in Figure 3C, the capacity of colony formation in SMMC-7721/shAurora-A cells was significantly reduced in comparison with SMMC-7721/shcontrol cells. Then, the role of Aurora-A in chemotherapy-induced apoptosis in HCC cells was further determined. Results showed that ADR or CDDP-induced apoptosis of both SMMC-7721/shAurora-A and SMMC-7721/shcontrol cells was in a dose-dependent manner. However, the apoptotic rate of SMMC-7721/shAurora-A cells significantly increased, when compared with SMMC-7721/shcontrol cells at the same concentration of ADR or CDDP (Figure 3D). Western blotting analysis for cleaved caspase-3 demonstrated that SMMC-7721/shAurora-A cells had a considerably higher amount of cleaved caspase-3 than SMMC-7721/shcontrol cells upon the same concentration of ADR or CDDP treatment (Supporting Figure.1A). Therefore, silencing of Aurora-A significantly increases the *in vitro* chemosensitivity of HCC cells by enhancing chemotherapy-induced apoptosis.

**Figure 4:**
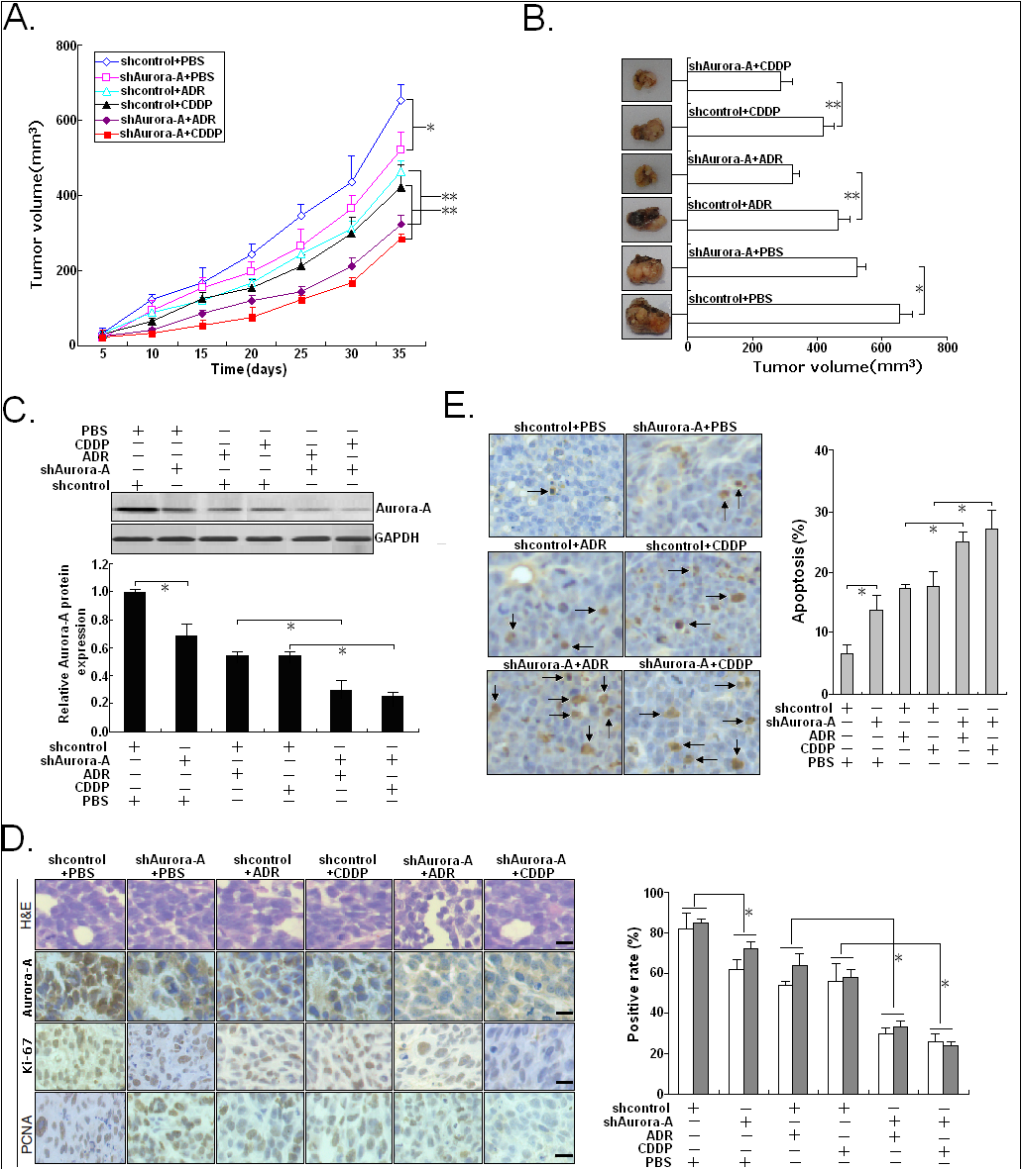
Effects of Aurora-A downregulation on *in vivo* chemosensitivity of HCC cells Mice were treated with ADR (2.0 mg/kg body weight; i.p., thrice), DDP (3.0 mg/kg body weight; i.p., thrice), or with 0.1ml PBS (pH 7.4; i.p., thrice). (A) Growth of tumors in the mice injected with SMMC-7721/shAurora-A or SMMC-7721/shcontrol cells treated with ADR, DDP or PBS. The inoculation was done in eight mice. (B) Representative features of tumors 35d after inoculation using SMMC-7721/shAurora-A or SMMC-7721/shcontrol cells treated with ADR, DDP or PBS. (C) Western blotting detection of Aurora-A protein expression in tumors developed from SMMC-7721/shAurora-A or SMMC-7721/shcontrol cells treated with ADR, DDP or PBS, respectively. GAPDH was used as an internal control. (D) Immunostaining of Aurora-A, Ki-67 and PCNA protein expression in tumors developed from SMMC-7721/shAurora-A or SMMC-7721/shcontrol cells treated with ADR, DDP or PBS. Upper: H&E staining; Intermediate and lower: immunostaining; Bars, 100μm. (E) TUNEL assay detection of apoptosis in tumors developed from SMMC-7721/shAurora-A or SMMC-7721/shcontrol cells treated with ADR, DDP or PBS, respectively. Data were presented as mean ± SD of at least three independent experiments. *N.S, P*>0.05; *P<0.05; **P<0.01.

Next, we further investigated the role of Aurora-A downregulation on the *in vivo* sensitivity of HCC cells to ADR or CDDP in a mice xenograft model. Then, s.c. tumors were formed in nude mice followed by treatment with ADR or CDDP. The tumors formed from SMMC-7721/shAurora-A were apparently smaller than those formed from SMMC-7721/shcontrol cells after the ADR or CDDP treatment at day 35 (Figure 4A). At 35 days after inoculation, the tumor volume was measured. Following the treatment with ADR or DDP, the average volumes of tumors formed from SMMC-7721/shAurora-A cells were significantly lower than those of tumors formed from SMMC-7721/shcontrol cells (Figure 4B). Following the treatment with ADR or CDDP, tumor homogenates were subjected to Western blotting detection of Aurora-A protein expression, and we showed that the expression of Aurora-A protein in xenografts formed from SMMC-7721/shAurora-A cells was significantly downregulated in comparison with that in xenografts formed from SMMC-7721/shcontrol cells (Figure 4C). Following the treatment with ADR or CDDP, immunohistochemistry was performed to detect the expression of Aurora-A, Ki-67 and PCNA. As shown in Figure 4D, the positivity of Aurora-A protein in xenografts from SMMC-7721/shAurora-A cells was significantly weaker than that in xenografts from SMMC-7721/shcontrol cells. Also, the number of Ki-67 or PCNA-positive cells in xenografts formed from SMMC-7721/shAurora-A cells was higher than that in xenografts from SMMC-7721/shcontrol cells. TUNEL assay was performed to detect apoptosis, and results showed that the rate of apoptotic tumor cells in xenografts formed from SMMC-7721/shAurora-A cells was higher than that in xenografts formed from SMMC-7721/shcontrol cells, following the treatment with ADR or CDDP (Figure 4E). These data indicate that silencing of Aurora-A significantly increases the *in vivo* chemosensitivity of HCC cells.

### Overexpression of Aurora-A reduces *in vitro* and *in vivo* chemosensitivity of HCC cells by preventing chemotherapy-induced apoptosis

**Figure 5:**
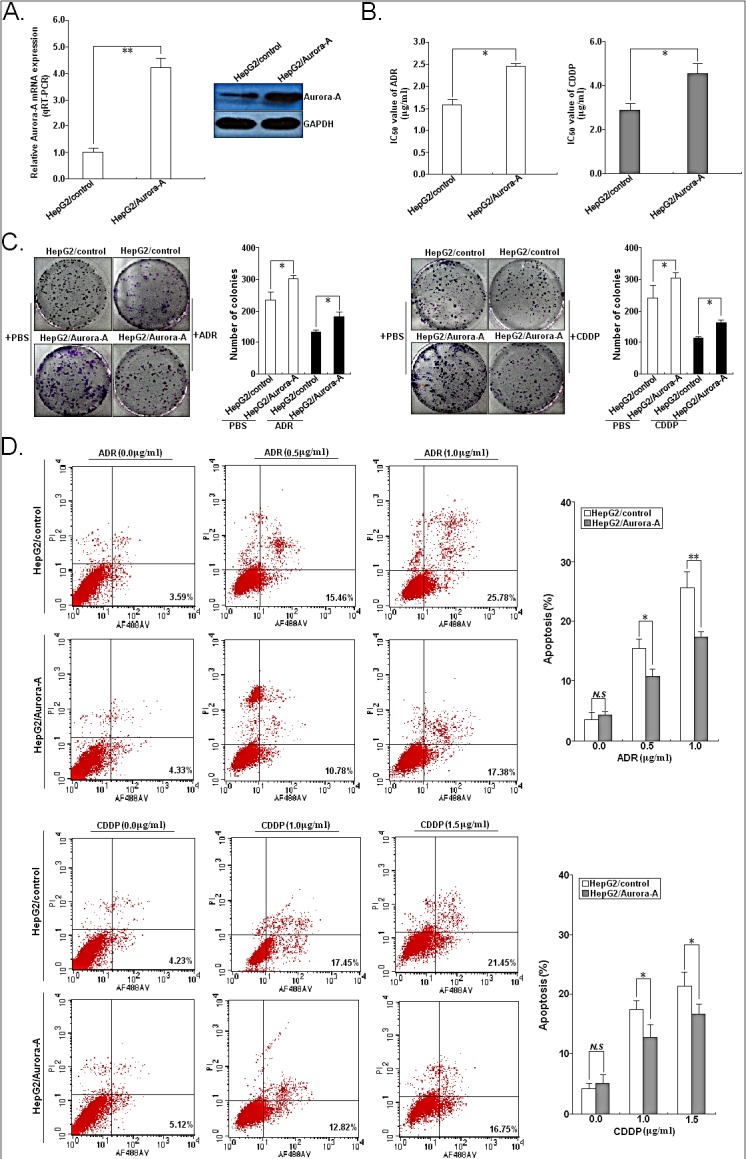
Effects of Aurora-A upregulation on *in vitro* chemosensitivity of HCC cells (A) qRT-PCR and Western blotting detection of Aurora-A mRNA and protein expression in stably transfected HepG2/Aurora-A or HepG2/control cells, respectively. GAPDH was used as an internal control. (B) MTT analysis of IC_50_ values of ADR or CDDP in HepG2/Aurora-A or HepG2/control cells, respectively. (C) The colony formation of HepG2/Aurora-A or HepG2/control cells treated with various concentrations of ADR (0.0 or 0.5 μg/ml) or CDDP (0.0 or 1.0 μg/ml). (D) Flow cytometric analysis of apoptosis in HepG2/Aurora-A or HepG2/control cells combined with various concentrations of ADR (0.0, 1.0 and 2.0 μg/ml) or CDDP (0.0, 1.5 and 3.0 μg/ml). Data are expressed as the mean±SD of three individual experiments. *N.S, P*>0.05; *P<0.05; **P<0.01.

We next examined whether Aurora-A overexpression reduces chemosensitivity of HCC cells via stale transfection of pMD/Auro construct into HepG2 cells (Figure.5A). Then, the chemosensitivity of those stable cells was determined by *in vitro* assays. Results indicated that the IC_50_ values of both ADR and CDDP were significantly increased by Aurora-A overexpression in HepG2 cell line (Figure.5B). The IC_50_ value of ADR or CDDP in HepG2/Aurora-A cells was 2.45±0.28 or 4.56±0.41 μg/ml (*P*<0.05), respectively, while the IC_50_ value of ADR or CDDP in HepG2/shcontrol cells was 1.58±0.44 or 2.86±0.36 μg/ml, respectively. Then, we determined the effect of Aurora-A overexpression on the colony formation ability of HCC cells when exposed to ADR (0.0 or 0.5 μg/ml) or CDDP (0.0 or 1.0 μg/ml) treatment, and results indicated that the capacity of colony formation in HepG2/Aurora-A cells was significantly increased compared with HepG2/control cells (Figure 5C). The *in vitro* effects of Aurora-A overexpression on HCC cell apoptosis of HepG2 was determined. As shown in Figure 5D, the apoptotic rate of HepG2/Aurora-A cells significantly reduced, when compared with HepG2/control cells at the same ADR or CDDP concentration. Western blotting analysis for cleaved caspase-3 demonstrated that HepG2/Aurora-A cells had a considerably lower amount of cleaved caspase-3 than HepG2/control cells upon the same concentration of ADR or CDDP treatment (Supporting Figure 1B). Therefore, overexpression of Aurora-A significantly reduces the *in vitro* chemosensitivity of HCC cells by reducing chemotherapy-induced apoptosis.

**Figure 6:**
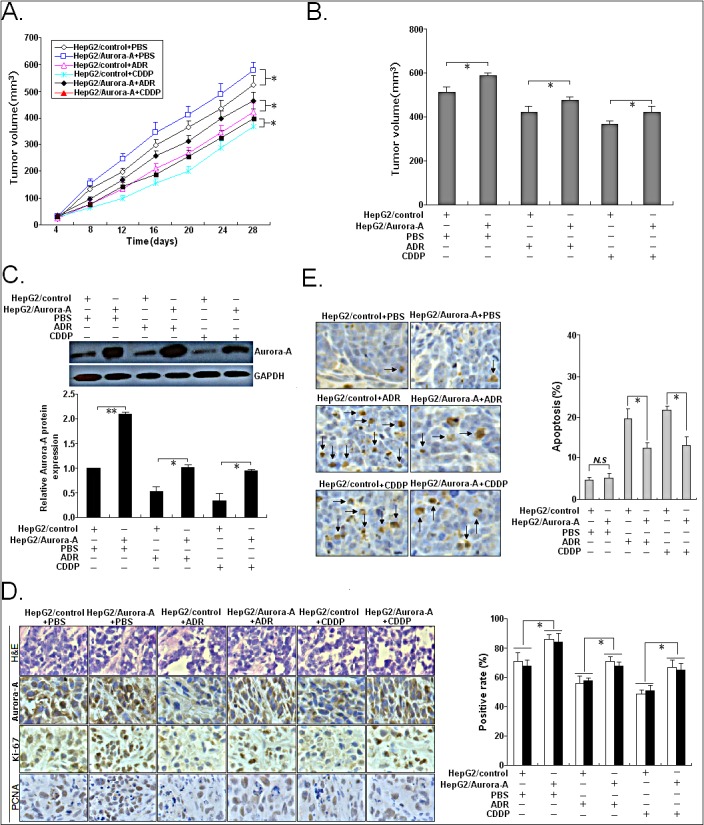
Effects of Aurora-A upregulation on *in vivo* chemosensitivity of HCC cells Mice were treated with ADR (2.0 mg/kg body weight; i.p., thrice), DDP (3.0 mg/kg body weight; i.p., thrice), or with 0.1ml PBS (pH 7.4; i.p., thrice). (A) Growth of tumors in the mice injected with HepG2/Aurora-A or HepG2/control cells treated with ADR, DDP or PBS. The inoculation was done in eight mice. (B) Representative features of tumors 28d after inoculation using SMMC-7721/shAurora-A or SMMC-7721/shcontrol cells treated with ADR, DDP or PBS. (C) Western blotting detection of Aurora-A protein expression in tumors developed from HepG2/Aurora-A or HepG2/control cells treated with ADR, DDP or PBS, respectively. GAPDH was used as an internal control. (D) Immunostaining of Aurora-A, Ki-67 and PCNA protein expression in tumors developed from HepG2/Aurora-A or HepG2/control cells treated with ADR, DDP or PBS. Upper: H&E staining; Intermediate and lower: immunostaining; Bars, 100μm. (E) TUNEL assay detection of apoptosis in tumors developed from HepG2/Aurora-A or HepG2/control cells treated with ADR, DDP or PBS, respectively. Data were presented as mean ± SD of at least three independent experiments. *N.S, P*>0.05; *P<0.05; **P<0.01.

Then, we further investigated the effects of Aurora-A overexpression on the *in vivo* chemosensitivity of HCC cells. Then, s.c. tumors were formed in nude mice following the treatment with ADR or CDDP. The tumors formed from HepG2/Aurora-A were apparently bigger than those formed from HepG2/control cells after the ADR or CDDP treatment at day 28 (Figure 6A). Following the treatment with ADR or DDP, the average volume of tumors formed from SMMC-7721/shAurora-A cells was significantly lower than that of tumors formed from SMMC-7721/shcontrol cells at 28 days after inoculation (Figure 6B). Following the treatment with ADR or CDDP, Western blotting detection of Aurora-A protein expression indicated that the expression of Aurora-A protein in xenografts formed from HepG2/Aurora-A cells was significantly upregulated in comparison with that in xenografts formed from HepG2/control cells (Figure 6C). Following the treatment with ADR or CDDP, immunohistochemistry showed that the positivity of Aurora-A protein in xenografts from HepG2/Aurora-A cells was significantly stronger than that in xenografts from HepG2/control cells. Also, the number of Ki-67 or PCNA-positive cells in xenografts formed from HepG2/Aurora-A cells was lower than that in xenografts from HepG2/control cells (Figure 6D). TUNEL assay indicated that the rate of apoptotic tumor cells in xenografts formed from HepG2/Aurora-A cells was lower than that in xenografts formed from HepG2/control cells, following the treatment with ADR or CDDP (Figure 6E). Therefore, overexpression of Aurora-A significantly reduces the *in vivo* chemosensitivity of HCC cells.

### The miR-21/PTEN signaling was involved in Aurora-A-promoting chemoresistance of HCC cells

**Figure 7:**
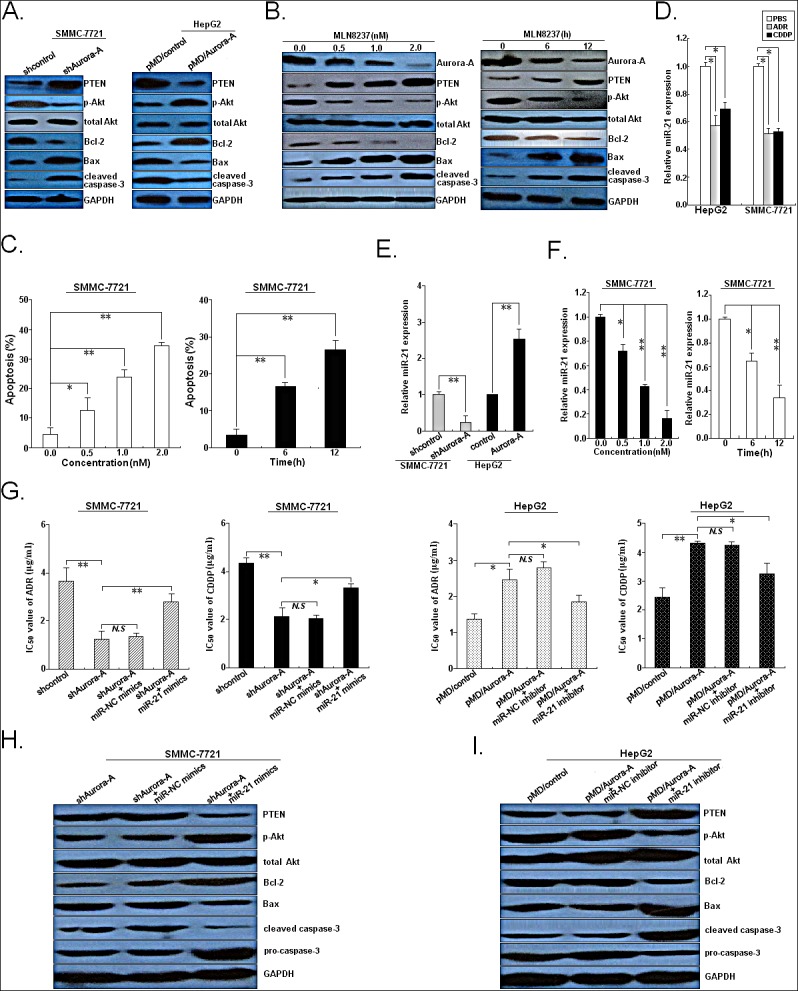
Aurora-A inhibits chemotherapy-induced apoptosis in HCC cells by upregulation of miR-21 (A) Western blotting detection of apoptosis-related proteins (PTEN, p-Akt, total Akt, Bcl-2, Bax, cleaved caspase-3 and total caspase-3) in SMMC-7721/shAurora-A (or SMMC-7721/shcontrol) or HepG2/Aurora-A (or HepG2/control) cells. (B) Western blotting detection of total Aurora-A, p-Aurora-A and above apoptosis-related proteins in SMMC-7721 cells treated with various concentrations of MLN8237 (0.0, 0.5, 1.0 and 2.0 nM) for 24h or lengths (0, 6 and 12h) of MLN8237 (1.5 nM). (C) Flow cytometry detection of apoptosis in SMMC-7721 cells treated with various concentrations of MLN8237 (0.0, 0.5, 1.0 and 2.0 nM) for 24h or lengths (0, 6 and 12h) of MLN8237 (1.5 nM). (D) 24h after HCC cells (HepG2 and SMMC-7721) were treated with PBS, ADR (0.5 μg/ml) or CDDP (1.0 μg/ml), qRT-PCR detection of miR-21 expression. (E) qRT-PCR detection of miR-21 expression in SMMC-7721/shAurora-A (or SMMC-7721/shcontrol) or HepG2/Aurora-A (HepG2/control) cells, respectively. (F) qRT-PCR detection of miR-21 expression in SMMC-7721 cells treated with various concentrations of MLN8237 (0.0, 0.5, 1.0 and 2.0 nM) for 24h or lengths (0, 6 and 12h) of MLN8237 (1.5 nM). (G) MTT analysis of IC_50_ values of ADR or CDDP in SMMC-7721/shAurora-A or SMMC-7721/shcontrol cells or co-transfection with miR-21 or miR-NC mimics, or in HepG2/Aurora-A or HepG2/control or or co-transfection with miR-21 or miR-NC inhibitor, respectively. (H) Western blotting detection of above apoptosis-related proteins in SMMC-7721/shAurora-A cells or co-transfection with miR-21 or miR-NC mimics. (I) Western blotting detection of above apoptosis-related proteins in HepG2/Aurora-A cells or co-transfection with miR-21 or miR-NC inhibitor. GAPDH or U6 was used as an internal control. Data were presented as mean ± SD of at least three independent experiments. *N.S, P*>0.05; *P<0.05; **P<0.01.

PTEN acts as a tumor suppressor gene through the action of its phosphatase protein product, and it negatively regulates the PI3K/Akt pathway, which directly affects the apoptosis by targeting Bcl-2 family proteins [11]. The above mentioned results indicated that ADR or CDDP treatment could markedly decrease expression of Aurora-A protein and increase expression of PTEN protein, and therefore we determined the effect of Aurora-A on expression of PTEN, phospho-Akt (p-Akt), total Akt, Bcl-2, Bax and cleaved caspase-3 proteins (Figure 7A). The expression level of PTEN protein in SMMC-7721/shAurora-A cells was significantly higher than that in SMMC-7721/shcontrol cells (*P*<0.05), while the expression level of PTEN protein in HepG2/Aurora-A cells was significantly lower than that in HepG2/control cells (*P*<0.05). Meanwhile, silencing of Aurora-A could lead to the decreased expression of p-Akt and Bcl-2 proteins in SMMC-7721 cells and the increased expression of Bax and cleaved caspase-3 proteins in HepG2 cells, while overexpression of Aurora-A could induce the opposite effects in HCC cells. Treating cells with various dosages (0.0, 0.5, 1.0 and 2.0 nM) and lengths (1.5 nM: 0, 6 and 12 h) of MLN8237 (a selective Aurora A inhibitor), which resulted in a loss of phospho-Aurora-A (p-Aurora-A) but not total Aurora-A, markedly increased PTEN expression and also significantly decreased p-Akt levels in SMMC-7721 cells (Figure 7B). Also, we showed that there was a marked dose- and time-dependent decrease in Bcl-2 protein expression and increase in the expression of Bax and cleaved caspase-3 proteins in cells treated with MLN8237 (Figure 7B). Furthermore, treating cells with various dosages of MLN8237 could induce a marked dose- and time-dependent decrease in apoptosis of SMMC-7721 cells (Figure 7C).

It has been reported that aberrant expression of miR-21 can contribute to HCC growth and spread by modulating PTEN expression and PTEN-dependent pathways involved in mediating phenotypic characteristics of cancer cells such as cell growth, migration, and invasion [12]. Also, miR-21 induces resistance to the anti-tumour effect of interferon-α / 5-fluorouracil in HCC cells [13]. Thus, whether Aurora-A induces miR-21 expression to regulate PTEN expression and promotes chemoresistance in HCC cells is unknown. First, we showed that pretreatment with ADR (0.5 μg/ml) or CDDP (1.0 μg/ml) for 24h led to decreased expression of miR-21 in HCC cells (Figure 7D). Then, we detect the effect of Aurora-A expression on the expression of miR-21 in HCC cells. Silencing of Aurora-A led to the decreased expression of miR-21 in SMMC-7721 cells, while overexpression of Aurora-A induced the increased expression of miR-21 in HepG2 cells (Figure 7E). Also, treating cells with various dosages of MLN8237 could induce a marked dose- and time-dependent decrease in miR-21 expression in SMMC-7721 cells (Figure 7F). To further confirm target specificity between miR-21 and PTEN in HCC cells, SMMC-7721 or HepG2 cells was transiently transfected with miR-21 inhibitor (or miR-NC inhibitor) or miR-21 mimics (or miR-NC mimics), respectively. It was observed that silencing of miR-21 could lead to the increased PTEN protein expression and upregulation of miR-21 could lead to the decreased PTEN protein expression in HCC cells (Supporting Figure 2A and B). Then, we performed luciferase reporter assay with a vector containing the putative PTEN 3′-UTR target site downstream of the luciferase reporter gene. Luciferase activities of SMMC-7721 cells transfected with PTEN-wt construct were significantly lower after transfection of miR-21 mimics and were significantly higher after transfection of miR-21 inhibitor, whereas those with PTEN-mut construct showed no significant difference (Supporting Figure 2C). Results of flow cytometry indicated that the apoptosis of SMMC-7721 cells transfected with miR-21 inhibitor was significantly higher than that of cells transfected with miR-NC inhibitor (*P*<0.01; Supporting Figure 2D). Also, silencing of miR-21 could significantly increase the expression of apoptosis-promoting proteins (PTEN, Bax, cleaved caspase-3) and decrease the expression of apoptosis-inhibiting proteins (p-Akt, Bcl-2 and pro-caspase-3) (Supporting Figure 2E). Furthermore, downregulation of miR-21 significantly increased the sensitivity of SMMC-7721 cells to ADR or CDDP by enhancing apoptosis, while overexpression of miR-21 significantly decreased the sensitivity of HepG2 cells to ADR or CDDP by reducing apoptosis (Supporting Figure 2F and G).

Next, we determined the roles of miR-21 in Aurora-A-promoting chemoresistance of HCC cells. Upregulation of miR-21 could partially rescue the decreased IC_50_ values of ADR or CDDP in SMMC-7721 cells induce by Aurora-A downregulation, while silencing of miR-21 could partially rescue the increased IC_50_ values of ADR or CDDP in SMMC-7721 cells induce by Aurora-A downregulation (Figure 7G). Meanwhile, upregulation of miR-21 could partially reverse the increased apoptosis and expression of PTEN/Akt signaling-related proteins of SMMC-7721 cells induced by Aurora-A downregulation (Figure 7H). Also, silencing of miR-21 could partially reverse the expression of PTEN/Akt signaling-related proteins of HepG2 cells induced by Aurora-A overexpression (Figure 7I). Collectively, these data demonstrated that the miR-21/PTEN signaling was involved in Aurora-A-promoting chemoresistance of HCC cells by regulating caspase-3-dependent apoptosis.

### MiR-21 promoter is activated by NF-κB in response to Aurora-A overexpression in HCC cells

**Figure 8:**
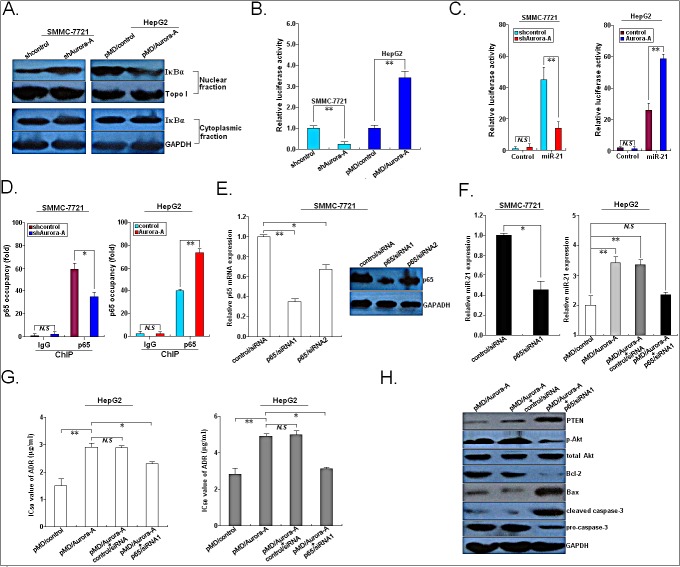
Aurora-A upregulates miR-21 transcription via NF-κB in HCC cells (A) Western blotting detection of nuclear or cytoplasmic IκBα protein expression in SMMC-7721/shAurora-A (or SMMC-7721/shcontrol) or HepG2/Aurora-A (or HepG2/control) cells. Topo I or GAPDH was used as an internal control, respectively. (B) The activity of NF-κB was measured in SMMC-7721/shAurora-A (or SMMC-7721/shcontrol) or HepG2/Aurora-A (or HepG2/control) cells using a luciferase reporter system. (C) The promoter of miR-21 was activated by Aurora-A. SMMC-7721/shAurora-A (or SMMC-7721/shcontrol) or HepG2/Aurora-A (or HepG2/control) cells were treated with pGL3/miR-21-promoter or pGL3-Basic, respectively. (D) ChIP assays with anti-NF-κB/p65 antibodies showed binding of NF-κB to the promoter of miR-21 in SMMC-7721/shAurora-A (or SMMC-7721/shcontrol) or HepG2/Aurora-A (or HepG2/control) cells. The relative occupancies of NF-κB/p65 are indicated as vertical bars. The bar graphs show the averages of three independent ChIP experiments. (E) qRT-PCR and Western blotting detection of p65 mRNA and protein expression in SMMC-7721 transiently transfected with control/siRNA, p65/siRNA1 or p65/siRNA2, respectively. (F) qRT-PCR detection of miR-21 expression in SMMC-7721 cells transiently transfected with control/siRNA or p65/siRNA1, or HepG2/Aurora-A (or HepG2/control) cells or co-transfection with control/siRNA or p65/siRNA1, respectively. U6 was used as an internal control. (G) MTT analysis of IC_50_ values of ADR or CDDP in HepG2/Aurora-A (or HepG2/control) or co-tranfection with control/siRNA or p65/siRNA1, respectively. (H) Western blotting detection of above apoptosis-related proteins in HepG2/Aurora-A cells or co-transfection with control/siRNA or p65/siRNA1, respectively. GAPDH was used as an internal control. Data were presented as mean ± SD of at least three independent experiments. *N.S, P*>0.05; *P<0.05; **P<0.01.

Next, we elucidated the underling molecular mechanisms involved in Aurora-A-inducing activation of miR-21. Previously, it has been reported that Aurora-A induces phosphorylation of IκBα, thereby mediating its degradation and loss of IκBα leads to activation of NF-κB target gene transcription [14]. Additionally, activation of NF-κB is sufficient to activate the expression of miR-21 [15]. Therefore, we hypothesized that Aurora-A overexpression might induce the increased miR-21 expression by inducing NF-κB activation. First, we determined the effects of Aurora-A expression on the expression of IκBα in the cytoplasmic and nuclear fractions of HCC cells, and showed that silencing of Aurora-A could lead to the increased expression level of nuclear IκBα protein in SMMC-7721 cells and overexpression of Aurora-A could lead to the decreased expression level of nuclear IκBα protein in HepG2 cells (Figure 8A). Then, SMMC-77211 or HepG2 cells were co-transfected with a NF-κB-dependent luciferase reporter plasmid (2×NF-κB-Luc) together with pSil/shAurora-A (or pSil/shcontrol) or pMD/Aurora-A (or pMD/control), respectively. Assays of luciferase activity in lysates indicated that silencing of Aurora-A reduced NF-κB activity in SMMC-7721 cells and overexpression of Aurora-A increased NF-κB activity in HepG2 cells, when compared with respective control vector (Figure 8B). Further luciferase assay showed that silencing of Aurora-A reduced the promoter activity of miR-21 in SMMC-7721 cells and overexpression of Aurora-A increased the promoter activity of miR-21 in HepG2 cells (Figure 8C). ChIP assay indicated that NF-κB/p65 could bind to the promoter region of miR-21, which could be blocked in SMMC-7721 cells by silencing of Aurora-A and enhanced in HepG2 cells by overexpression of Aurora-A (Figure 8D). Then, we further showed that silencing of p65 significantly downregulated the expression of miR-21 in SMMC-7721 cells and could reverse the increased miR-21 expression in HepG2 cells induced by Aurora-A overexpression (Figure 8E and F). Furthermore, silencing of p65 could not only partially reverse the increased IC_50_ values of ADR or CDDP but also partially restore the decreased apoptosis-promoting proteins (PTEN, Bax, cleaved caspase-3) and the increased expression of apoptosis-inhibiting proteins (p-Akt, Bcl-2 and pro-caspase-3) in HCC cells induced by Aurora-A overexpression (Figure 8G and H). These data clearly suggest that NF-κB activation is required for miR-21 upregulation in response to Aurora-A overexpression in HCC cells.

### Expression of Aurora-A was positively correlated with miR-21 expression but negatively correlated with PTEN in HCC tissues

**Figure 9:**
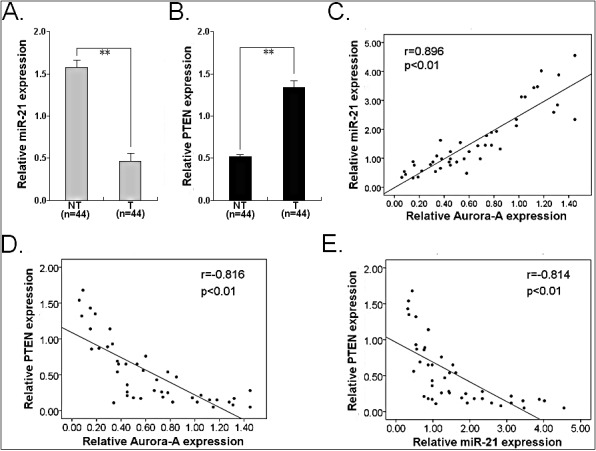
Expression of miR-21 and PTEN protein and their correlations with Aurora-A protein expression in HCC tissues (A) qRT-PCR detection of miR-21 expression in 44 paired of HCC and NTs. (B) Western blotting detection of PTEN protein expression in 44 paired of HCC and NTs. (C) A statistically significant positive correlation between miR-21 and Aurora-A protein expression levels in 44 cases of LAD tissues (Spearman's correlation analysis, r = 0.896; *P*<0.01). (D) A statistically significant inverse correlation between Aurora-A and PTEN protein expression levels in 44 cases of LAD tissues (Spearman's correlation analysis, r = −0.816; *P*<0.01). (E) A statistically significant inverse correlation between miR-21 and PTEN protein expression levels in 44 cases of LAD tissues (Spearman's correlation analysis, r = −0.814; *P*<0.01). Data were presented as mean ± SD of at least three independent experiments. Corresponding *P* values analyzed by Spearman correlation test are indicated.

To further analyze the correlations between Aurora-A, miR-21 and PTEN in HCC, we performed qRT-PCR and Western blot assays to detect the expression of miR-21 and PTEN protein in 44 pairs of primary HCC and corresponding NTs. It was observed that the relative level of miR-21 expression in HCC tissues was significantly higher than that in corresponding NTs (*P*<0.01; Figure 9A). Also, the mean level of PTEN protein in HCC tissues was significantly lower than that in corresponding NTs (*P*<0.01; Figure 9B). In the same HCC tissues, we then correlated Aurora-A protein with the expression levels of miR-21 and PTEN protein. Significant positive correlation was observed when miR-21 expression level was plotted against Aurora-A protein expression level (2-tailed Spearman's correlation, r = 0.896, *P*<0.01) (Figure 9C), while significant inverse correlation was observed when PTEN protein expression level was plotted against Aurora-A protein expression level (2-tailed Spearman's correlation, r = −0.816, *P*<0.01) (Figure 9D). In addition, a significant inverse correlation was observed when miR-21 expression level was plotted against PTEN expression level (2-tailed Spearman's correlation, r = −0.814, *P*<0.01) (Figure 9E). These data further support the existence of a novel Aurora-A/ NF-κB/miR-21/PTEN signaling in HCC.

## DISCUSSION

Aurora-A is an important member of a new serine/threonine kinase family, which is involved in mitotic entry, separation of centriole pairs, accurate bipolar spindle assembly, and alignment of metaphase chromosomes and completion of cytokinesis [16]. It has been reported that Aurora-A overexpression can lead to genetic instability (aneuploidy) which may cause malignant transformation in many tissues [17]. The overexpression of Aurora-A has been found in a variety of human cancer and correlated with poor prognosis of patients. Tanaka' et al reported that the up-regulation of Aurora-A expression may reflect the malignant behavior of ESCC and prove useful information as a prognostic factor for ESCC patients [18]. Likewise, Yang' et al showed that Aurora-A is amplified and overexpressed in ESCC from Chinese patients [7]. Also, Reiter and his colleagues showed that the up-regulation of AURKA mRNA may play a critical role in the tumor progression of head and neck squamous cell carcinoma and provides useful information as a prognostic factor for patients [19]. The correlations of Aurora-A overexpression with poor prognosis of patients are also reported in other human cancers, including epithelial ovarian cancer, laryngeal squamous cell carcinoma, and breast cancer, etc [20-22]. Jeng' et al first reported the overexpression and amplification of Aurora-A in HCC, and further showed that overexpression of Aurora-A was correlated with high grade and high stage of patients [23]. Then, in our previous study, we employed semi-quantitative RT-PCR assay to evaluate the correlation of Aurora-A mRNA expression and prognosis of HCC patients, and showed that high Aurora-A mRNA expression was significantly correlated with advanced tumor stage, more frequent lymph node or hematogenous metastasis, higher incidence of cancer-related death and poorer prognosis of HCC patients. However, the clinicopathological or prognostic significance of Aurora-A protein in HCC is still fully understood. First, we performed Western blotting assay to detect the expression of Aurora-A in 44 paired of HCC and the adjacent nontumor tissues, and showed that the averaged level of Aurora-A protein in HCC tissues was higher than that in the adjacent nontumor tissues, which was confirmed by results of immunohistochemistry analysis. It was also found that high Aurora-A protein expression was more frequently to be detected in tumors with advanced TNM stage and higher incidence of lymph node metastasis. Together with the above and previous evidence, it was thus proposed that overexpression of Aurora-A may play critical roles in HCC progression and metastasis. Kaplan-Meier analysis of survival indicated that HCC patients with high Aurora-A protein expression tend to have worse RFS and OS in comparison with patients with low Aurora-A protein expression. Multivariate Cox model analysis indicated that increased Aurora-A protein expression was a marker of poor overall survival independent of adjusted factors, suggesting that Aurora-A might be a molecular prognostic marker additive to TNM stage for HCC patients. These results were in consistent with investigations in other types of human cancers. Therefore, the negative correlation between Aurora-A overexpression and better prognosis of HCC patients may be used for identifying HCC patients who are more likely to have tumor relapse in clinical practice, thus, good candidates to receive more aggressive treatment.

Increasing evidence indicates that Aurora-A regulates malignant phenotypes of tumor cells. It has been reported that overexpression of Aurora-A could promote growth and inhibit apoptosis of tumor cells. For example, Wang' et al provided evidence that Aurora-A overexpression promotes cell proliferation and inhibits apoptosis, suggesting a novel mechanism that is closely related to malignant phenotype and anti-cancer drugs resistance of ESCC cells [24]. The same research group showed that stable knockdown of Aurora-A by vector-based RNA interference in human ESCC cell line inhibits tumor cell proliferation and enhances apoptosis, which makes a promising therapeutic strategy for the treatment of ESCC and other malignant tumors overexpressing Aurora-A [25]. In gastric cancer, Sehdev and his colleagues showed that Aurora-A could promote tumor growth and cell survival through regulation of HDM2-induced ubiquitination and inhibition of P53 [26]. At the same time, the same research group found Aurora-A to be up-regulated during chronic inflammation to promote activation of NF-κB and tumorigenesis in analyses of gastric cancer cell lines, human tissue samples, and mouse models [27]. Targeting Aurora-A can suppress the growth of other types of human tumor cells, including oral squamous cell carcinoma, glioblastoma and small cell lung cancer [28-30]. Additionally, the correlations of Aurora-A expression with tumor invasion and metastasis are increasingly reported. Do' et al showed that Aurora kinase A mediates epithelial ovarian cancer cell migration and adhesion [31]. Wang and his colleagues reported that overexpression of Aurora-A contributes to the malignancy development of ESCC by enhancing tumor cell invasion as well as MMP-2 activity and expression, which can occur through signaling pathways involving p38 MAPK and Akt protein kinases [32]. Meanwhile, it was reported that Aurora-A can induce mammary cell migration and breast cancer metastasis by activating the Cofilin-F-actin pathway [33]. Importantly, the roles of Aurora-A overexpression in chemo- or radioresistance of tumor cells are also reported. Hata' et al showed that RNA interference targeting aurora kinase a suppresses tumor growth and enhances the taxane chemosensitivity in human pancreatic cancer cells [34]. Tanaka' et al reported that the suppression of aurora-A/STK15/BTAK expression enhances chemosensitivity to docetaxel in human ESCC [35]. Borges and his colleagues showed that inhibition of Aurora kinases enhances chemosensitivity to temozolomide and causes radiosensitization in glioblastoma cells [36]. Likewise, these data were in consistent with investigations in other types of human malignancies, including renal cell carcinoma, lung cancer and prostate cancer [37-39]. These data suggest that Aurora-A might be a promising molecular target for chemo- or radiosensitizing human cancers. In our previous study, we have shown that siRNA targeting Aurora-A suppresses proliferation and induces apoptosis in human HCC cells [10]. Our further research indicated that Aurora-A might be a key regulator of HIF-1α-promoting malignant phenotypes of HCC by activation of Akt and p38-MAPK signaling pathways [40]. Benten' et al showed that Aurora kinase inhibitor PHA-739358 suppresses growth of HCC *in vitro* and in a xenograft mouse model [41]. The roles of Aurora-A overexpression in HCC progression are obvious, but the correlation of Aurora-A expression with chemosensitivity of HCC cells is still unclear. To testify it, we analyzed the correlations of the level of Aurora-A protein expression with the sensitivity of HCC cells to ADR or CDDP, and found that the expression level of Aurora-A is positively associated with chemosensitivity of HCC cells. Meanwhile, ADR or CDDP treatment could induce the decreased expression of Aurora-A and anti-apoptotic protein Bcl-2 protein, and the increased expression of PTEN, pro-apoptotic protein Bax and cleaved caspase-3. Then, we further investigated the effect of Aurora-A expression on chemosensitivity of HCC cells. First, SMMC-7721 cells (high-Aurora-A) were stably transfected with pSil/shAurora-A, and qRT-PCR and Western blotting assays confirmed the downregulation of Aurora-A. It was observed that silencing of Aurora-A could lead to the decreased IC_50_ values of ADR and CDDP by enhancing chemotherapy-induced caspase-3-dependent apoptosis. Meanwhile, the *in vivo* chemosensitivity of HCC cells could also be significantly increased by Aurora-A downregulation. Likewise, HepG2 cells (low-Aurora-A) were stably transfected with pMD/Auro, and upregulation of Aurora-A could significantly decrease the chemosensitivity of HCC cells both *in vitro* and *in vivo*. Therefore, overexpression of Aurora-A promotes formation of chemoresistant phenotype in HCC cells.

Next, the molecular mechanisms involved in Aurora-A-promoting chemoresistance in HCC cells will be explored. The phosphatase and tensin homologue (PTEN) deleted on the chromosome 10 gene was cloned by association with the human cancer susceptibility locus at 10q23 and is a lipid phosphatase that dephosphorylates phosphatidylinositol 3,4,5-trisphosphate (PIP3) to PI(4,5)P2 and opposes the PI3K/AKT pathway, exerting tumour suppressor activity [42]. It has been reported that loss of PTEN can lead to activation of the AKT signaling pathway. In this study, we demonstrated that silencing of Aurora-A could lead to the decreased protein expression of PTEN, p-Akt, Bcl-2, pro-caspase-3 and the increased protein expression of Bax and cleaved caspase-3. At the same time, overexpression of Aurora-A could lead to the opposite effects on those proteins. Importantly, treating SMMC-7721 cells with various dosages and lengths of MLN8237 (a selective Aurora A inhibitor) not only induce caspase-3-dependent apoptosis but also induces upregulation of PTEN and Bax, downregulation of Bcl-2 and inhibition of Akt phosphorylation in a concentration - and time - dependent manner. It is well known that activation without PTEN mutations is likely to occur in many human cancers, and the regulation of PTEN can be regulated through multiple mechanisms, including transcription, mRNA stability, microRNA targeting, translation and protein stability [43]. Recently, non-coding small RNAs such as miRNAs were reported to play important roles in a variety of cellular processes such as growth, differentiation, motility and malignant transformation [44]. A single miRNA can have multiple targets involved in different oncogenic pathways, while a single gene can also be regulated by several miRNAs. Such miRNAs are often aberrantly expressed during cancer development and subject to the regulation by signaling pathways key to cancer development. Here, we described a novel signaling pathway in which miR-21 can negatively control PTEN activation in HCC cells upon Aurora-A upregulation. MiR-21 functions as an oncogenic microRNA by targeting multiple tumor suppressor genes including PTEN, PDCD4, BCL-2, TPM1, and RECK, and its participation has been reported in many human cancers [45-49]. PTEN was reported as a direct target of miR-21 that was involved in miR-21-mediated effects on tumor biology: cell growth, migration, and invasion in human HCC. The dysregulation of miR-21/PTEN signaling has been found in regulation of malignant phenotypes in other human malignancies, such as breast cancer, glioma, lung cancer, etc [50-52]. Thus, we will explore whether Aurora-A negatively regulates PTEN expression in HCC cells by regulating miR-21 expression. Here, we demonstrated that silencing of Aurora-A could lead to the decreased expression of miR-21 and upregulation of Aurora-A induced the increased expression of miR-21 in HCC cells. Also, inhibition of miR-21 significantly increased ADR or CDDP-induced apoptosis in HCC cells, while upregulation of miR-21 significantly reduced chemotherapy-induced apoptosis. Further research indicated that miR-21 mimics could partially reverse the decreased IC_50_ values of ADR or CDDP and changes of PTEN/Akt signaling-related proteins in SMMC-7721 cells induced by Aurora-A downregulation and miR-21 inhibitor partially reverse those changes in HepG2 cells induced by Aurora-A upregulation. These data suggest that Aurora-A promotes chemoresistance in HCC cells by regulation of miR-21/PTEN/Akt signaling.

The biogenesis of miRNAs from gene transcription to posttranscriptional maturation is subject to regulation by different signaling pathways important to carcinogenesis. A computational screen revealed that NF-κB was located in miR-21 gene transcriptional element, and the transcriptional regulation NF-κB was located in miR-21 gene transcriptional element, and transcriptional regulation of miR-21 by NF-κB have been reported in many human cancers, such as breast cancer, glioma, and gastric cancer [53-55]. Therefore, we investigated whether Aurora-A-induced NF-κB was bind to the binding sites of miR-21 and activated miR-21 to downregulate PTEN expression in HCC cells. Previously, it has been reported that upregulation of Aurora-A could increase NF-κB activity in tumor cells. In epithelial ovarian cancer cells, Aurora-A is reported to induce phosphorylation of IκBα and its subsequent cytoplasmic degradation and leads to constitutive NF-κB activity, which creates a pro-inflammatory and anti-apoptotic environment [56]. Briassouli and his colleagues defined a new role for Aurora-A in regulating IκBα that is critical for the activation of NF-κB-directed gene expression and may be partially responsible for the oncogenic effect of Aurora-A when the gene is amplified and overexpressed in human tumors [14]. In this study, we showed that Aurora-A promotes expression of nuclear IκBα protein and enhances NF-κB activity in HCC cells. Further ChIP assay indicated that NF-κB/p65 could binding to the promoter region of miR-21, which could be blocked or enhanced by Aurora-A downregulation or overexpression. Also, suppression of NF-κB could mimic the effects of silencing of Aurora-A or miR-21 on chemosensitivity of HCC cells by targeting PTEN/Akt signaling, and attenuated Aurora-A-enhanced miR-21 expression, further suggesting that Aurora-A activates NF-κB to promote chemoresistance in HCC cells by targeting miR-21/PTEN/Akt signaling pathway. Of course, although Aurora-A-induced expression of miR-21 was regulated by NF-κB, but we cannot rule out the possibility that Aurora-A could induce additional miRNAs that could directly or indirectly target PTEN/Akt signaling or that targeting other molecular signaling pathways, so we will investigate those questions further in future research.

Taken together, this study demonstrated that the expression of Aurora-A protein was significantly upregulated in HCC cell lines and tissue samples. Patients with positive Aurora-A protein had shorter RFS and OS than those with negative Aurora-A protein, and multivariate Cox model analysis indicated that positive Aurora-A immunostaining was an independent prognostic factor for HCC patients. As the number of patients in this study is limited, a prospective study with a larger number of patients is required to evaluate the prognostic significance of Aurora-A immunostaining in HCC. Silencing of Aurora-A significantly increases chemosensitivity of HCC cells, while overexpression of Aurora-A significantly decreases chemosenistivity of HCC cells. Furthermore, overexpression of Aurora-A reduces chemotherapy-induced apoptosis by promoting NF-κB-mediated transcription of miR-21, which negatively regulates PTEN and then inhibits caspase-3-mediated apoptosis induction. This novel Aurora-A/NF-κB/miR-21/PTEN axis provides new insight into the mechanisms underlying HCC chemoresistance, and target this signaling pathway may be a potential therapeutic strategy for chemosensitization of HCC in the future.

## MARTERIALS AND METHODS

### Patients and tissue samples

Primary HCC and corresponding nontumor liver tissues were obtained from 44 patients who received primary HCC resection at the Liver Disease Center of the 81th Hospital of PLA and the Department of Hepatobiliary Surgery of First Hospital Affiliated to the Chinese PLA General Hospital between March 2005 and March 2007. All tumors were histopathologically confirmed to contain at least 80% malignant cells and none of the participants received preoperative treatment. The tumor type and the grade of cell differentiation were designated based on the criteria of World Health Organization (WHO), whereas the pathological stage of each tumor was determined by the International Union Against Cancer (UICC) TNM classification. Except tissues used for RNA extraction, the remnant tissues were rapidly frozen in liquid nitrogen and stored at −80°C. Ethical approval was obtained from the hospital and fully informed consent from all patients prior to sample collection. This study was approved by the Review Board of Hospital Ethics Committee (Jingling Hospital, Nanjing University).

### Cell culture and chemotherapeutic reagents

Two human HCC cell lines (SMMC-7721 and HepG2) were purchased from Shanghai Institute of Cell Biology (Shanghai, China). All cell lines were cultured in RPMI 1640 (GIBCO-BRL) medium supplemented with 10% fetal bovine serum (FBS), 100 U/ml penicillin, and 100 μg/ml streptomycin in humidified air at 37°C with 5% CO_2_. Adriamycin (ADR) and cisplatin (CDDP) and MLN8237 (Aurora-A inhibitor) were purchased from Promega Co. (Madison, Wisconsin, USA). Stock solutions of ADR (2.0 μg/mL) and CDDP (3.0 μg/mL) were prepared with DMSO and diluted with PBS to the required concentrations before each experiment.

### Plasmid construction and cell transfection

Short hairpin RNA (shRNA) specifically targeting human Aurora-A (GenBank no. NM_003600) was designed to knockdown Aurora-A expression. The shRNA sequences targeting Aurora-A and negative control shRNA are as follows: shAurora-A, 5′-*GATCCATGCCCTGTCTTAACTGTCATTCAAGAGAT GACAGTAAGACAGGGCATAGA-*3′; shcontrol: 5′-*GATCCAAGCTGAAGTACAACCTTCTTCAAGAGAGA AGGTTGTACTTCAGCTTAGA*-3′. All the above sequences were inserted into the BglII and HindIII enzyme sites of pSilencer4.1-CMVneo vector, respectively. The recombinant plasmids were named pSil/shAurora-A and pSil/shcontrol, respectively. The recombinant vectors were confirmed by the digestion analysis of restriction endonuclease, and all the constructed plasmids were confirmed by DNA sequencing. The plasmid vector (pMD18/Auro) expressing open-reading frame of Aurora-A was purchased from Sino Biological Inc (Beijing, P.R. China). MiR-21 mimics (miR-NC mimics), miR-21 inhibitor (miR-NC inhibitor) or p65/siRNA1, p65/siRNA2 (or control/siRNA) were synthesized by Genepharma (Shanghai, China). The sequences of siRNAs were as follows: p65/siRNA1: 5′-*GCCCTATCCCTTTACGTCA*-3′; p65/siRNA2: 5′-*TCCTTTCAGGAGATGAAGA*-3′; control/siRNA: 5′-*ACTCTATCTGCACGCTGAC*-3′. The cell transfection was performed in opti-MEM with the transfection reagent Lipofectamine™ 2000 (Invitrogen, CA, USA) following the manufacturer's instructions. For stable transfection, the cell lines transfected with pSil/shAurora-A (or pSil/shcontrol) or pMD/Auro (or pMD/control) vector were stably selected with G418 (400 mg/mL) 48 h later after transfection, and individual clones were isolated and maintained in a medium containing G418 (100 mg/mL).

### Western blotting assay

Cell protein lysates were separated in 10% SDS polyacrylamide gels, electrophoretically transferred to polyvinylidene difluoride membranes (Roche). Protein loading was estimated using mouse anti-GAPDH monoclonal antibody. Lab Works^TM^ Image Acquisition and Analysis Software (UVP, Upland, CA, USA) were used to quantify band intensities. Antibodies were purchased from Univ-bio Inc (Shanghai, China). The cells were lysed and p222rotein extraction was done. The samples were separated in 10% SDS acrylamide gel and electrophoretically transferred to polyvinylidene difluoride membrane (Thermoscitific, American). The membrane was blotted with 10% non-fat milk, washed and then probed with the rabbit anti-human Aurora-A (1:100 dilution), phospho-Aurora-A (p-Aurora-A) (1:100 dilution), PTEN (1:150 dilution), Bcl-2 (1:50 dilution), Bax (1:50 dilution), cleaved caspase-3 (1:200 dilution), pro-caspase-3 (1:100 dilution), p65 (1:150 dilution) and GAPDH (1:100 dilution), followed by treatment with secondary antibody conjugated to horseradish peroxidase. The proteins were detected by the enhanced chemiluminescence system and exposed to x-ray film. All antibodies were purchased from Univ-bio Inc (Shanghai, China).

### RNA extraction and real-time quantitative RT-PCR (qRT-PCR) assay

Total RNA was extracted from the tissues or cultured cells using Trizol (Invitrogen, CA, USA) according to the manufacturer's protocol, reverse transcribed using Taqman™ microRNA reverse transcription kit and subjected to real-time PCR using TaqMan™ MicroRNA Assay kit (Applied Biosystems, USA) according to the manufacturer's instructions. Reactions were performed using Stratagene Mx3000 instrument in triplicate. MiRNA expression was normalized to U6 snRNA. The qRT-PCR primers of miR-21, U6, Aurora-A, p65 and GAPDH were obtained from the Harvard Primer Bank, as listed in Supporting Table 5. Reactions were performed using ABI 7500 fast RT- PCR system instrument in triplicate.

### *In vitro* chemosensitivity assay

The single-cell suspensions were prepared and dispersed in 96-well plates. After incubation for 72 h with the ADR or CDDP compounds, the 3-(4,5-Dimethyl-2-thiazolyl)-2,5-diphenyl-2H-tetrazolium bromide (MTT) assay (Sigma, USA) solution (0.5 mg/ml) was added. Following incubation for 4 h, 100 μL of extraction buffer were added to each well. After an overnight incubation, absorbance at 490 nm was measured using using a microplate reader (Bio-Rad, Model 680).

### *In vivo* chemosensitivity assay

Animal studies were performed according to institutional guidelines. The mock or stably transfected HCC cells were suspended in 100 μL PBS and injected subcutaneously into the right side of the posterior flank of female BALB/c athymic nude mice (Department of comparative medicine, Jinling Hospital, Nanjing, China) at 5 to 6 weeks of age. When the average tumor size reached about 50 mm^3^, ADR or CDDP was given through intraperitoneal injection with a concentration of 2.0 mg/kg (ADR) or 3.0 mg/kg (CDDP), one dose every other day with 3 doses totally. Tumor growth was examined weekly for at least 5 wk. After 35 days, the mice were killed, necropsies were performed, and tumors were weighted. Tumor volumes were calculated by using the equation V (in mm^3^)=*A*×*B*^2^/2, where *A* is the largest diameter, and *B* is the perpendicular diameter.

### Colony formation assay

Cells were trypsinized to single cell suspensions and were seeded 6-well plates at 500/well. After 14 days culture RPMI 1640 medium, the colonies were stained with Giemsa solution and the number of colonies was counted. Each experiment was performed in triplicate.

### Flow cytometric detection of apoptosis

An annexin V-fluorescein isothiocyanate (FITC) apoptosis detection kit (Oncogene Research Products, Boston, MA) was used to detect apoptosis according to the manufacturer's instructions. All of the samples were assayed in triplicate.

### Immunohistochemistry assay

Transplanted tumor tissues were immunostained for Aurora-A, Ki-67 and PCNA. The signal was amplified and visualized using 3, 30-diaminobenzidine chromogen followed by counterstaining with hematoxylin. Expression was considered positive when 50% or more of cancer cells were stained. Immunohistochemical analysis was done to detect Aurora-A protein in human LA tissues. Formalin-fixed, paraffin-embedded tissue was freshly cut. Sections were incubated in a moist chamber with primary rabbit anti-human Aurora-A monoclonal antibody (Santa Cruz Biotechnology,. Santa Cruz, CA, USA) for 30 min at room temperature, followed by a secondary antibody for 30 min (DakoCytomation, Denmark). Rabbit serum was used as negative control. The staining intensity of Aurora-A protein was scored 0, 1+, 2+, 3+, or 4+, and the samples with scores up to 2+ were considered as positive and the samples with other scores were considered as negative.

### TUNEL assay

The apoptosis in transplated tumor tissues was also monitored by Terminal deoxynucleotidyl transferase-mediated dUTP nick end labeling TUNEL method. The TUNEL assay was performed in according to the guidelines recommended of the (TUNEL) assay kit (KeyGen, Nanjing, China).

### Luciferase activity

The cells grown in a 48-well plate were cotransfected with miRNA mimics/inhibitors, and pLUC firefly luciferase vectors containing empty, wild-type or mutant PTEN 3′-UTR sequence using lipofectamine 2000 (Invitrogen, Carlsbad, CA, USA). NF-κB-dependent luciferase reporter plasmid (2×NF-κB-Luc) and miR-21 promoter luciferase reporter plasmid (pGL3/miR-21-promoter) were provided by Dr Ming Jun. Luciferase activity assays for miRNA target validation were performed 30 and 48h post transfection, respectively, using luciferase activities using the Dual-Luciferase Assay kit (Promega, USA). Relative luciferase activity was calculated by normalizing the ratio of Firefly/Renilla luciferase to that of negative control-transfected cells.

### Electrophoretic Mobility Shift Assay (EMSA)

To detect the DNA binding activity of NF-kB, electrophoretic mobility shift assay was done following the manufacturer's instructions (Panomics, Inc., Redwood City, CA) as previously described [57]. Briefly, nuclear proteins were prepared using a nuclear extraction kit and their concentrations determined by protein estimation procedure. A biotin-labeled NF-kB probe with a 5′-*AGTTGAGGGGACTTTCCCAG-*

*GC*-3′ sequence or an unlabeled cold probe was used to bind nuclear proteins at 15 to 20 °C for 30 min. Products were run on a 6% nondenaturing polyacrylamide gel in 0.5×Trisborate EDTA at 120V for 60 min at 4°C; the shifted bands corresponding to the protein/DNA complexes were separated relative to the unbound dsDNA. The gel was then transferred onto a presoaked membrane at 300 mA for 30 min at 4°C. Following the immobilization of bound oligonucleotides in the membrane by a UV-crosslinking oven for 5 min, the shifted bands were visualized after exposure to film.

### Chromatin immunoprecipitation (ChIP) assay

ChIP assay was performed with Immunoprecipitation Assay Kits (Millipore, USA) according to the manufacturer's instructions. Briefly, cells were cross-linked with 1% formaldehyde for 10 min at 37°C. The cells were then resuspended in 200 μl of lysis buffer and incubated for 10 minutes on ice. The lysate was sheared to lengths between 200 and 1000 base pairs by sonication. The supernatant was pre-cleared with a Salmon Sperm DNA/Protein A Agarose-50% Slurry. The recovered supernatant was incubated with anti-p65 antibody (Abcam, HongKong) or an isotype control IgG overnight at 4°C with rotation. Then, the antibody/DNA complex was collected using Salmon Sperm DNA/Protein A Agarose Slurry for one hour at 4°C with rotation. The complex was eluted by elution buffer. Then, the crosslinks were reversed with 5M NaCl heating at 65°C for 4 hours. The DNA sample was then purified and measured by Q-PCR.

### Statistical analysis

All statistical analyses were performed using the SPSS 17.0 software package (SPSS, Chicago, IL, USA). Experimental data were expressed as the mean ± SEM. For comparison of means between two groups, a two-tailed *t*-test was used, and for comparison of means among three groups, one-way ANOVA was used The Spearman correlation test was used for analyses of primary tumors. Survival probabilities were determined using Kaplan-Meier analysis and the significance of difference was analyzed by a log-rank test. A Cox proportional hazards regression analysis was used for multivariate analyses of prognostic values. Significance was accepted at *P*<0.05.

## SUPPLEMENTARY MATERIAL, FIGURES AND TABLES



## Abbreviations

HCC: hepatocelluar carcinoma
microRNA-21: miR-21
NF-kappaB: NF-κB
Iκβα: Ikappaβ-alpha, Iκβα;
siRNA: small interfering RNA
ADR: adriamycin
CDDP: cisplatin
shRNA: short hairpin RNA
PTEN: phosphatase and tensin homolog deleted on chromosome ten
RFS: recurrence-free survival
OS: overall survival
