# Supplementary material for: Aurora-A promotes chemoresistance in hepatocelluar carcinoma by targeting NF-kappaB/microRNA-21/PTEN signaling pathway
Source: Oncotarget. 2014 Nov 4;5(24):12916–35. doi: 10.18632/oncotarget.2682 (PMC4350360; doi:10.18632/oncotarget.2682)
Supplement: Supplementary file 1 [file oncotarget-05-12916-s001.pdf]

Aurora-A promotes chemoresistance in hepatocelluar carcinoma by targeting NF-kappaB/microRNA-21/PTEN signaling pathway

Supplementary Material

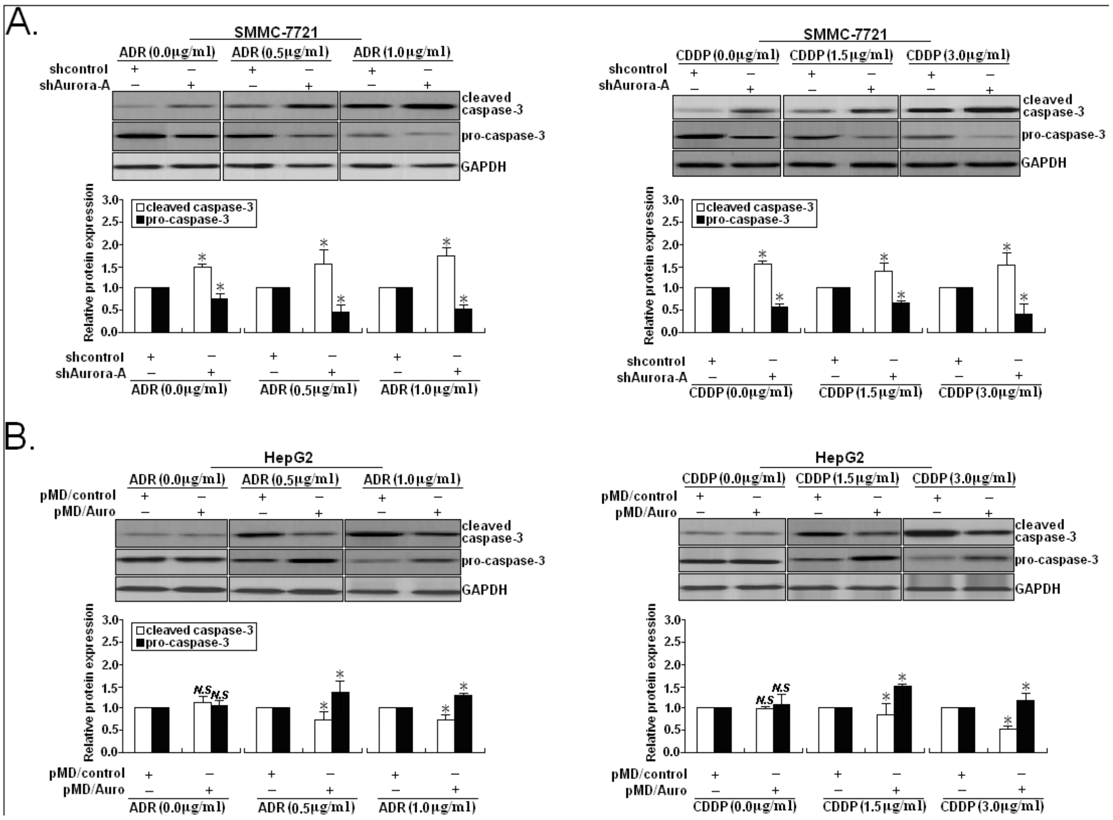

Supporting Figure 1: Effects of Aurora-A expression on cleaved or total caspase-3 protein expression in HCC cells treated with ADR or CDDP. (A) Western blotting detection of cleaved caspase-3 or total caspase-3 protein expression in SMMC-7721/shAurora-A or SMMC-7721/shcontrol cells treated with various concentrations of ADR (0.0, 1.0 and 2.0 μg/ml) or CDDP (0.0, 1.5 and 3.0 μg/ml). (B) Western blotting detection of cleaved caspase-3 or total caspase-3 protein expression in SMMC-7721/shAurora-A or SMMC-7721/shcontrol cells treated with various concentrations of ADR (0.0, 0.5 and 1.0 μg/ml) or CDDP (0.0, 1.0 and 1.5 μg/ml). GAPDH was used as an internal control. Data were presented as mean ± SD of at least three independent experiments. *N.S*, $P>0.05$; $^*P<0.05$.

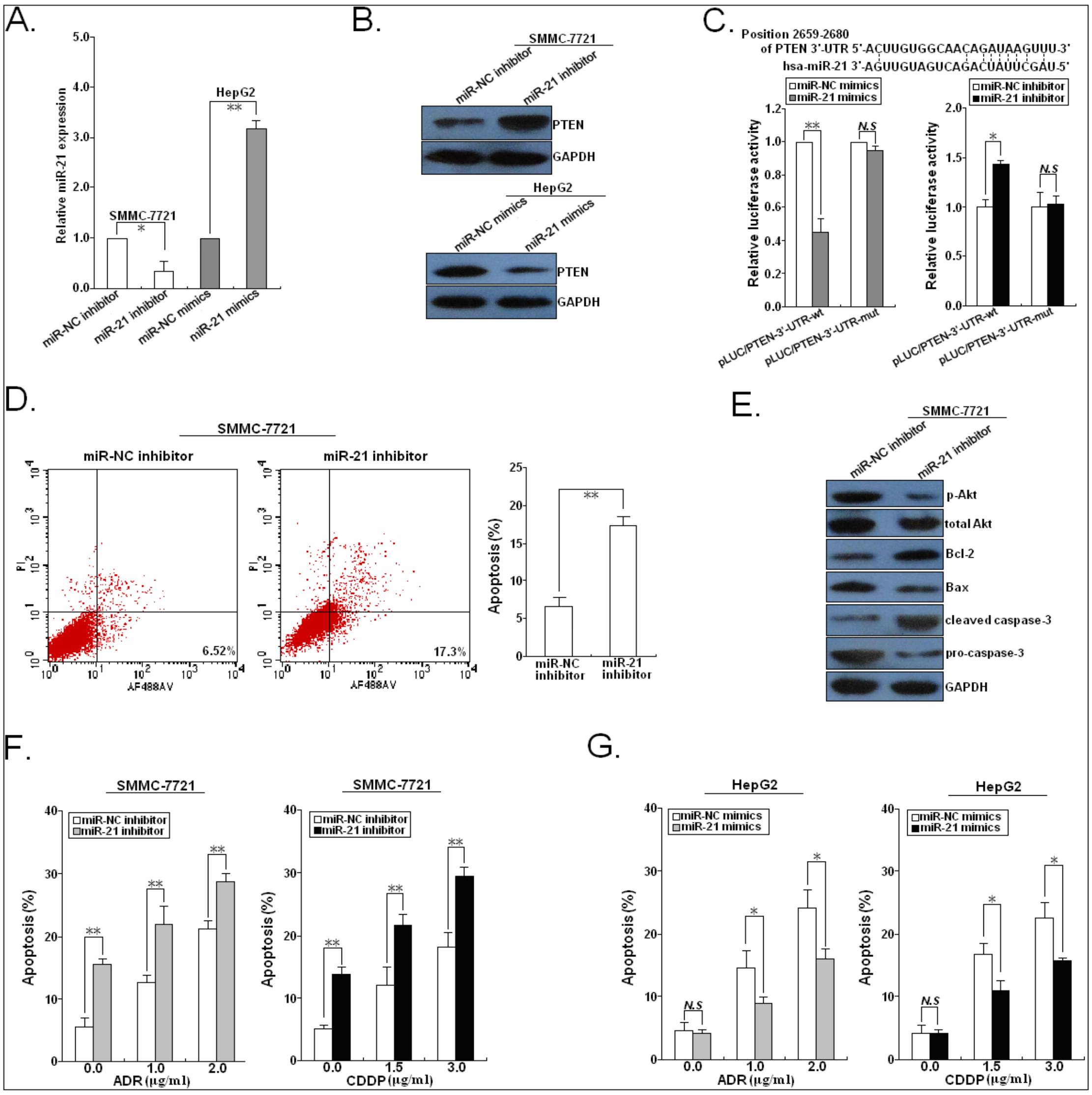

Supporting Figure 2: PTEN was a functional target of miR-21 in HCC cells. (A) qRT-PCR detection of miR-21 expression in miR-21 inhibitor (or miR-NC inhibitor)-transfected SMMC-7721 cells or miR-21 mimics (or miR-NC mimics)-transfected HepG2 cells. U6 was used as an internal control. (B) Western blotting detection of PTEN protein expression in miR-21 inhibitor (or miR-NC inhibitor)-transfected SMMC-7721 cells or miR-21 mimics (or miR-NC mimics)-transfected HepG2 cells. GAPDH was used as an internal control. (C) Relative luciferase activity was analyzed after wildtype (pLUC/PTEN-3'-UTR-wt) or mutant (pLUC/PTEN-3'-UTR-mut) 3'-UTR reporter plasmids were co-transfected with miR-21 (or miR-NC) mimics or miR-21 (or miR-NC) inhibitor in HepG2 cells. The histogram shows the mean±SD of the normalized luciferase activity from three independent experiments. (D) Flow cytometry detection of apoptosis in miR-21 or miR-NC inhibitor-transfected SMMC-7721 cells. (E) Western blotting detection of the expression of p-Akt, total Akt, Bcl-2, Bax, cleaved caspase-3 and total caspase-3 proteins in miR-21 or miR-NC inhibitor-transfected SMMC-7721 cells. GAPDH was used as an internal control. (F) Flow cytometry detection of apoptosis in miR-21 or miR-NC inhibitor-transfected SMMC-

7721 cells treated with various concentrations of ADR (0.0, 1.0 and 2.0 μg/ml) or CDDP (0.0, 1.5 and 3.0 μg/ml). (G) Flow cytometry detection of apoptosis in miR-21 or miR-NC mimics-transfected HepG2 cells treated with various concentrations of ADR (0.0, 0.5 and 1.0 μg/ml) or CDDP (0.0, 1.0 and 1.5 μg/ml). Data were presented as mean ± SD of at least three independent experiments. *N.S*, $P>0.05$; *$P<0.05$; **$P<0.01$.

Supporting Table 1. Aurora-A protein expression levels were detected by Western blotting in 44 pairs of HCCs and corresponding nontumor liver tissues (NTs).

Tissue Group	Total No. of Tissues	No. with Aurora-A overexpression	Ratio (%)	*P*-value
HCC	44	32	72.7	<0.001
NT	44	8	18.2	

Supporting Table 2. Aurora-A protein expression levels were detected by immunohistochemistry assay in 44 pairs of HCCs and corresponding nontumor liver tissues (NTs).

Tissue Group	Total No. of Tissues	No. with Aurora-A overexpression	Ratio (%)	*P*-value
HCC	44	26	59.1	<0.001
NT	44	12	27.3	

Supporting Table 3. Correlation of Aurora-A with clinicopathologic characteristics of HCC patients.

Characteristics	Aurora-A immunostaining		*P*-value
	Positive (n=28)	Negative (n=16)	
Gender			0.728
Female	16	10	
Male	12	6	
Age (years)			0.572
<55	5	4	
≥55	23	12	
Alchohol intake			0.463
No	10	4	
Yes	18	12	
Liver function			0.864
Child-Pugh A	13	7	
Child-Pugh B	15	9	
AFP (ng/L)			0.820
≤400	15	8	
>400	13	8	
TNM stage			0.019*
I/II	9	11	
IIII	19	5	
Lymph node metastasis			
No	11	14	0.002*
Yes	17	2	
Edmondson grade			
I+II	12	7	0.954
III	16	9	

NOTE: AFP, alpha-fetoprotein; TNM, tumor-node-metastasis. *$P<0.05$.

Support Table 4. Multivariate survival analyses of 5-year RFS and OS in HCC patients

HR: hazard ratio; 95% CI: 95% confidence interval. AFP, alpha-fetoprotein; TNM, tumor-node-metastasis. **P*<0.05.

		5-year RFS			5-year OS		
**Factor**	**Unfavorable/favorable**	*P*-value	HR	95% CI	*P*-value	HR	95% CI
Gender	Male / Female	0.546	1.023	0.756-1.324	0.177	0.873	0.542-1.388
Age	≥55 / <55	0.298	1.456	0.695-2.123	0.430	1.278	0.818-1.708
Alchohol intake	Yes / No	0.404	1.333	0.812-1.787	0.182	0.782	0.459-1.027
Liver function	Child-Pugh B / Child-Pugh A	0.612	0.988	0.701-1.338	0.098	1.562	0.772-2.123
AFP (ng/L)	>400 / <400	0.202	0.786	0.559-1.108	0.227	1.662	0.911-1.899
TNM stage	III / I+II	0.016*	2.128	1.878-3.025	0.008*	1.354	1.022-2.118
Lymph node metastasis	Yes / No	0.006*	1.802	1.234-2.797	0.035*	2.033	1.568-3.827
Edmondson grade	III / I+II	0.028*	1.777	1.188-3.085	0.108	1.611	0.809-2.177
Aurora-A protein expression	Positive / Negative	0.012*	2.055	1.735-3.455	0.005*	1.554	1.232-2.598

Supporting Table 5: Primers of qRT-PCR

Name	Primer
miR-21	RT: 5'-*GTCGTATCCAGTGCAGGGTCCGAGGTATTCGCACTGGATA CGACTCAACA*-3'
	F: 5'-*GTGCAGGGTCCGAGGT*-3'
	R: 5'-*GCCGCTAGCTTATCAGACTGATGT*-3'
U6	RT: 5'-*AACGCTTCACGAATTTGCGT*-3'
	F: 5'- *CTCGCTTCGGCAGCACA*-3'
	R: 5'-*AACGCTTCACGAATTTGCGT*-3'
Aurora-A	F: 5'-*AATGATTGAAGGTCGGATGC*-3'
	R: 5'-*TTCTCTGAGCATTGGCCTCT-3'*
p65	F: 5'-*TCTCCCTGGTCACCAAGGAC*-3'
	R: 5'-*TCATAGAAGCCATCCCGGC*-3'
GAPDH	F: 5'-*CCAAAAGGGTCATCATCTCTGC*-3'
	R: 5'-*TGCTAAGCAGTTGG TGGTGCAG*-3'

RT: reverse transcriptase; F: forward; R: reverse
